# The Bradshawe Lecture on Some Rare and New Diseases

**Published:** 1883-01

**Authors:** James Paget


### Selections.

THE BRADSHAWE LECTURE ON SOME RARE AND NEW DISEASES. Delivered at the Royal College of Surgeons of England on December 13th, 1882. By SIR JAMES PAGET, F.R.S.

*Mr. President and Gentlemen* :—It is my first duty, in delivering the first Bradshawe Lecture in our College, to offer a tribute of respectful thanks to the generous lady by whom it was founded, the widow of Mr. William Wood Bradshawe, a Fellow of this College, who practiced at Andover and at Reading, and died in 1866. He was a home-loving and studious man, who diligently cultivated his mind in both literature and science, and his widow, who survived him fourteen years, being desirous to testify her gratitude for the happiness which she owed to him, bequeathed a thousand pounds to this College, and as much to the Royal College of Physicians, on the condition that each should institute a lecture, to be given annually, and to bear his name. She desired that the lecture should be on some subject connected with medicine or surgery, and that the choice of the lecturer should rest with the President of each College for the time being. She made no stringent regulations, and seems to have wished only to maintain her husband's name in good repute by associating it with the advancement of the science which he loved.

In my endeavor to fulfill her examplary wish, I have chosen the subject of "Some Rare and New Diseases." I hope to be able, in speaking of them, to illustrate a part of the natural history of disease which I think is too little studied—that part, namely, which relates to the variations and the combinations of diseases in hereditary transmission. Besides, both in the choice of its subject and in the whole enterprise of giving this lecture, I have looked for an opportunity of promoting pathology by promoting pathological museums, a motive which I am sure will be pardoned though I am conscious of its being in some measure personal for I have spent so much time and thought in museums that I feel as if, in their greater utility, I should myself become more useful.

Now, first, respecting rare diseases, there may seem no want of opportunities of studying them. Our journals and the proceedings of our societies are full of the records of rare cases; many collections of such cases have been published, and there are many rare specimens in every museum. All these have that kind of attraction which belongs to everything that excites our wonder, but we too seldom let the wonder have its proper consequences; we too seldom let it provoke our curosity so far as to make us search for the meaning and reason of the rarity. There is a question we should often ask ourselves—Why is any disease rare? At least, why is any rare which does not depend on some accident or some rarely occuring extreme cause? I shall try to suggest answers which may be, in some intances, sufficient; but I fear that, in more instances, if I can be useful at all, it can only be by suggesting how answers may be found.

First, there is a difference, though it may seem only a verbal one, between rare cases and rare diseases. A case may be called rare when, though it is evidently one of a common disease, it differs from the usual type or standard of that disease in some one or two features. Thus it is a rare case when a common disease is found in an ususual place; as an epithelial cancer on the upper lip, or this fatty tumor on a finger: or in unusual quantity, as in this large cartilaginous tumor on a femur; or again, a case may be rare in respect of the time of its occurence. For instance, I have lately seen cancer of the rectum ending fatally in a lad of eighteen, and scrofulous abscess in a man of eighty; and many of us must have seen instances, though they are rare (and these are very important in the history of diseases), in which manifestations of syphilitic inheritance, usually evident in infancy, have not appeared till the time of youth or even of adult age; or cases may be very rare in respect of accidental complications or of the absence of some usual symptom. But of all these and other rare cases the number and variety are so great that it would impossible to deal generally with them, except as with mere story-telling. It would be very useful if someone would collect hundreds or thousands of them, and arrange them, even though it were only under such headings as I have just indicated. But even as they are singly and in disorder, let me say that we ought not to set them aside with idle thoughts or idle words about "curiosities" or "chances." Not one of them is without a meaning; not one but might be the beginning of of excellent knowledge, if only we could answer the question, Why is this rare? or, being rare, why did it in this instance happen?

But, because of their number and variety, I must pass by rare cases and will speak only of some rare diseases—that is, of some diseases which are rarely seen, and yet occur in a sufficient number of cases, and with sufficient uniformity, and sufficient difference from other diseases, to permit of their being described in general terms, and to justify their being called by distinctive names. And of these again, for they are numerous and various, I shall select that group which seems most attractive; the group of those, namely, of which there seems reason enough for believing—First, that they were, lately, new diseases and have become more frequent; and, secondly, that they are due mainly to morbid conditions changing and combining in transmission from parents to offspring. I say due mainly. It is certain that changes in the external conditions of our life have influence on even those morbid conditions which are most personal; but this influence is very hard or impossible to trace in the cases which I have in mind, and it may to-day be neglected though not forgotten. For, in all those cases, the personal factors and those of which alone I have to speak are more potent than the conditional, the inner than the outer. We call these diseases constitutional, diathetic, or by similar names; but the chief fact in them is that they, or the necessary previous states or predispositions to them, are inborn and inbred.

Let me first show that there is reason enough for believing that some rare diseases of this kind were recently—say within the last century—new; and that more recently, though still rare, they have become more frequent. There is, I know, a general unwillingness among pathologists to admit that there are new diseases of this kind; and this unwillingness is often just, for many diseases that may seem new have probably existed long and been overlooked; they may be new in knowledge, but not new in fact. Bright's disease and Addison's disease were new in the sense of having first been well observed and described by those whose names they bear; but no one would venture to say of diseases so difficult to detect, as these used to be, that they did not exist long before they were well observed. We could as well believe that embolism never occured till just before it was found out, or that right-side hemiplegia used not to be associated with aphasia. These things were old before they seemed to be new; but how long they had existed neither records nor museums can tell. It would be, indeed, very interesting if we could tell the time and manner of first appearing in the case of many diseases which are now common; but it is scarcely ever possible. And yet, if you will allow me a digression, let me show what in some instances museums may supply, and what I hope they will in the future supply much more largely. Here are specimens of typhoid ulcers of the intestines preserved by Hunter. Few things have been more important in the knowledge of fevers than the clear proofs of the distinction between typhus and typhoid given by Sir William Jenner in 1850. It was one of the best life-saving discoveries of this century; before it both diseases were at least partially misunderstood, and neither was so well treated as now. Since the distinction between them was discovered it has been possible to trace in old records cases probable instances of both; but there is nowhere so clear evidence of the occurence of typhoid a century or more ago as is given in these specimens of Hunter's, preserved without name or history; not unobserved, and yet not in any fair sense understood. Now in this, as in many things, Hunter set us a good example. He did not think these things unimportant which he did not understand. He was a thorough naturalist, and kept specimens of everything in his field of study which, though not yet, might become useful.

But, however much of what seems to be new we may justly ascribe to our previous oversight of what was old, there yet seems to be evidence enough that new diseases are in progress of evolution, and that, as I have said, some of the rare diseases of which I have to speak are the earliest instances of the new. Good evidence of this kind is to be found, I believe, in the peculiar joint disease discovered by M. Charcot in association with locomotor ataxy, and in the disease of bones to which I have given the name of osteitis deformans. Neither of these, I believe, was described till within the last few years. They may have been overlooked, but to believe this we must believe what is very improbable. We must believe that all the most acute and observant practitioners before our time overlooked, not merely obscure and transient diseases, difficult to study, but cases which lasted many years and gave constant great distress, and were manifested in signs so plain that they could recognized in the shape and gait, in the posture and whole aspect of the patients, in strangely large heads and curved limbs. And, further, we must believe that the morbid anatomists before ourselves overlooked changes of structure of the largest, most obvious, and most striking kind. It is, surely, very unlikely that they who studied and recorded such cases as those of extreme rickets and mollities ossium, and even called it rachitis adultorum, should have left unnoticed the cases of these two equally and somewhat similarly disfiguring and damaging diseases. This great improbability is strengthened by that which I believe to be a fact—that we have none but recently collected specimens of either of these diseases in our museums; not even among the crowds of bones and joints collected by our predecessors.

In twenty-six years I have seen twelve well-marked cases of the osteitis deformans, and about as many in which it was only partially evident. In the last six of these years I have seen seven of these cases, and others have been published, and yet I cannot find evidence that the disease was ever seen by any of those who had practice like my own; Brodie and Stanley, who saw as many cases of diseased bones as any surgeons of the last generation, had seen no case but which I showed them more than twenty-five years ago in the patient from whom these specimens were taken. Moreover, I cannot find on old specimen in our museums, or a representation of one in any book of plates, or a description of one in any catalogue. This might not seem very strange in the case of specimens troublesome or expensive to keep, or in such as are said to "show nothing." But these are very striking deviations from health, very plainly to be seen, and dry bones are neither costly nor troublesome to keep. We have large numbers of them collected by Hunter, Howship, Langstaff, Liston, Cooper, Stanley, and others, who collected not merely illustrations of diseases well known to them, but whatever was curious, whether it was understood or not. They would have looked on these bones as gems.

I might repeat this statement in nearly every particular concerning M. Charcot's disease. I believe there is not an old specimen in our museums. There is not one in the Musée Dupuytren; I cannot find a notice or an illustration of one. And yet this disease is now so far from being very rare that Dr. Buzzard has had nine cases under his eye at one time, and several have in recent years been shown in our societies.

Let me adduce one more instance of what I believe to have been new diseases within this century, though the museum evidence is not so strong as in those of which I have been speaking. Many believe, and, I think, quite rightly, that instances of typical gout, such as gained for it the name "podagra," have lately become comparatively rare, and that a large number of less acute diseases, regarded as forms of incomplete or supressed gout, are much more frequent. It may be that some or many of these lesser forms were always as common as they are now, but were overlooked or were not distinguished from other similar ailments. But here is a specimen of the effects of phlebitis of the femoral and external iliac veins, which, with its history, may tell that gouty inflammation of the veins was fifty years ago, if not a new disease, yet a much rarer one than it is now. Sir Henry Halford saw as much of gout, I suppose, as any man that ever lived; for he was for many years, during a very luxurious period, in the largest practice among the richest people in this town. He gave an account in 1832 of what he called phlegmasia dolens in the male. The disease so-called was common and well-known in women after parturition; so that, to justify his essay as a record of cases hitherto unobserved, it was enough for him to speak of phlegmasia dolens as occuring in men. He speaks of it as having been not long before regarded as "immediately occasioned by a deposit of milk;" but that "being tested by a more exact pathology," it was now atttributed to "an inflammation of the veins of the pelvis." And he says, "he was much mistaken if he had not seen three instances of it" in men "within the last few years." He then relates the case of the nobleman from whom, several years afterwards, this specimen was taken by Sir Astley Cooper—an admirable example of phlebitis, which we may be nearly sure was gouty, showing the changes in the blood-clots and in the walls of the veins during many years.

At the present time, phlebitis of this kind in the male can scarcely be called a very rare disease. There are few, I imagine, in large practice who have not seen many more than three cases within the last few years. So, we may believe, I think, that the disease has become more frequent in the last fifty years; and may suspect that not long before Sir Henry Halford's time it may have been a really new disease. It is hard to believe that it could have been overlooked. Its characters are strongly marked and evident to both eye and touch; it is a very painful, disabling, long-enduring disease, often recurring, sometimes observed in several members of the same family, and commonly leaving the affected limb large, heavy. and clumsy for many years. Could this have been overlooked when similar limbs, in consequence of an allied, though not the same disease, were known and described in women, and while, as it happened, the subject of phlebitis, in its traumatic and pyæmial forms was being very carefully studied? For there was a form of phlebitis much more common in the last century than in this; the phlebitis that occurred after bleeding. Hunter had studied this very carefully, had written on it, and shown it in these specimens; and after him, both this and the phlebitis after amputation were well-known. Especially after the beginning of this century the phlebitis after amputation was thoroughly worked at. It was only three years before the publication of Sir Henry Halford's essay that Mr. Arnott's renowned paper on inflammation of the veins was published in the Medico-Chirurgical Transactions. In the same volume are the chief papers by Dr. Robert Lee, on phlegmasia dolens; and he describes cases of phlebitis associated with pelvic cancer; but not one spontaneous phlebitis is mentioned by either of them.

Now, I think that in all these facts there is enough, not, indeed to prove, but to justify the belief that we have here examples of disease which have appeared in this country for the first time within the last century, and which have since become sufficiently frequent, and acquired sufficiently constant and distinctive characters to be described in general terms and called by new names. Let me repeat; these are not the diseases hard to be discerned. They are so well-marked, so distressing, so long-enduring, and both during life and after death so large and distinct in all their characters that it seems impossible that, unless they were very much rarer than they are now, they could have been overlooked.

I think it probable that there are other examples of the like kind; but I do not know them, and would rather go on to the second part of my subject—namely, to show the probability, or at least, to justify the hypothesis, that these diseases are among the instances of the results of morbid conditions, changing and combining in transmission from parents to offspring.

It should hardly be necessary to argue that changes of type in inherited diseases—changes which may be compared with the variations of species or of varieties in natural history—do take place. Yet I venture to think that many of us are prone to think too little of these variations; to regard them as rather unmeaning exceptions, or as the results of some unusual external conditions diverting diseases from their customary course.

It will be better for us if we study, in pathology as in natural history, varieties as much as species: changes as well as the more stable forms. Types of disease there are, standard forms, and the tenacity with which they are maintained—some, even, from pre-historic times—in all the varieties of the conditions of our lives is one of the most remarkable facts in all pathology. But they are not unalterable. Types vary in diseases, as in species; even in the diseases which depend least upon external conditions, and most on the qualities which are transmitted by inheritance.

Let me give some reasons why this must be.

1st. An exact likeness is never transmitted by inheritance; neither an exact likeness of either parent, nor an exact composite of both. This is evident enough in features, size, weight, and all that we can observe in external things. If we could be exactly endoscopic we should observe equal variation within; the same want of exact likeness in liver and lung, and, I venture to say, in blood and lymph and plasma, and whatever goes to make the whole person, healthy or diseased. The inheritance of likeness in disease, or liability to disease is, indeed, clear evidence of the transmission of likeness in the very minutest structure and composition. But the likeness is never perfect, it may in different persons deviate this way or that; it may vary towards disease or back again towards the healthy type; but it is never perfect, and in successive generations its degree of unlikeness may increase to a a great width of difference.

2nd. The certainty and probable extent of this variation must seem the greater if we consider the mingling of diathesis, and of all dispositions and liabilities to diseases in transmission from and through both parents. Consider the difficulty of maintaining the "breed" in any of the varieties of the species domesticated or cultivated by us, in horses or dogs, in pigeons or in seedling plants; the care that both parents should be of the same blood, or the same race, and that their produce should be raised in all due conditions; and then consider how numerous and wide, in spite of all this care, are the deflections from the type. With these facts before us we cannot imagine that diseased conditions should often be transmitted singly and unchanged; it is, surely, not likely that disease should be transmitted with more fixed conformity to type than normal compositions are. Hybrids and mongrels must be even more common among diseases than among species and varieties.

3rd. And, in thinking of the variation of diseases by combining or convergence of inherited qualities, we may not limit our thoughts to a single generation. It is reasonable to believe that instances occur of reversion, in which diseases or tendencies to disease may appear after a lapse of many generations. Such, I expect, are some of the cases in which leprosy has been seen, even of late years, in this country in persons never exposed to any of its external causes ; and to the like of this we may refer, I think, some of the rare cases which defy all efforts to refer them to any combination of types of disease now prevalent.

Now, I half wish that I could escape from the necessity of testing my doctrine by my facts ; but as I have often asked myself, so others may ask, how can the cases of rare disease of which I have been speaking, be explained as the results of morbid conditions changing and combining in transmission from parents to offspring. In the phlebitis we may often trace a variation from the customary type or standard of the very old and heritable disease, gout. In many cases its relations to typical gout are clear. The patients are members of gouty families, and in many of them other signs of gout are evident, either coincidently with the phlebitis or at other times ; it has, in short, all the evidences of being one of the many forms of what is called "incomplete gout." But, for a reason why this variety of gout settles (if I may so speak) in veins, especially in those of the lower extremities, I can only guess at a convergence of inherited dispositions both to a modified form of gout, and to some condition of veins rendering them, among all the structures, the most sensitive to the gouty process. Certainly it is not accident which determines the disease to the veins, for this disease "runs in families." I know of its occurrence in two brothers and three of their cousins ; and I have heard Sir Charles Locock tell of four sisters who had phlegmasia dolens, and whose father had crural phlebitis.

I am conscious that this is little more than guessing, and for the osteitis I must guess still further ; or, rather, let me say that, to the furthest bounds of propriety, I must exercise that use of the imagination which may happily discern a way towards the truth. I imagine, then, that a likeness of the osteitis deformans to several other diseases may indicate a combination, in definite proportions, of transmitted dispositions to those diseases; a combination which has become possible by changes of the type of one or more of them. First, it shows some relationship to mollities ossium and rickets, for, though it is an inflammatory disease, which they are not, yet the softening which permits of the curving of the bones is distinctive, and hardly occurs in any other form of inflammation of bone in middle or latter life. And, again, the relation of the osteitis to rickets and mollities ossium is notably indicated in the porous thickening of the skull, which is found in some instances of them all, and which is well marked in our specimens of genuine rickets from erroneous diet in young lions and young monkeys. Further, there appears some relation to gout, for some of the cases have known inheritance of gout, and instances are sometimes seen, in typically gouty persons, of a single bone having all the characters of the osteitis, though all the other bones appear healthy. Such a one is this femur, for the opportunity of showing which I am indebted to Mr. Bowlby, of St. Bartholomew's. There is a likeness, also, it may be said, to the osteo-arthritis and other forms of rheumatic gout in the remarkable maintenance of good general health during even many years of a painful and crippling inflammatory disease; and, further, there appears some relationship to cancer in the singular frequency with which cancer or sarcoma occurs in the healthy bones or other parts of those who have suffered for many previous years with osteitis deformans.

Thus, I imagine, by inherited dispositions, accumulating and combining or converging in definite proportions, this disease may be produced. I would try to imagine the genealogy of M. Charcot's disease, but that I have too little clinical knowledge of it. I can only suggest a combination of osteo-arthritis with syphilis chiefly localized in some spinal nerve center; but I believe far better suggestions may be made by those who, suspecting a combination of diseases rather than many radiating from one source, will carefully study the essays of Professor Charcot and Dr. Buzzard's admirable clinical lectures on Diseases of the Nervous System. Besides, I may seem to have guessed already more than enough. Let me, therefore, say that even if my guesses are wrong, my error cannot weaken the probability of the belief that these and rare diseases of like kind are instances of settled varieties of diseases, severally due to variations and convergence of morbid conditions in hereditary transmission. And if this be in any measure true, or even not more than a reasonable hypothesis, then it must be of great importance that we should know much more than we yet do of the variations which, in progress of time, diseases, or certain examples of them, may undergo; of their deviations in a gradually increasing number of instances from typical or standard forms; their acquirement in those instances of other comparatively fixed and long-abiding characters; of the occasional disappearance of old forms of disease, and the evolution of new ones. Such variations in diseases should be studied as Darwin studied the variations of species. Let me be clear in saying as Darwin studied; for in the pursuit of new knowledge he may be a model to all, as he has been to me so far as I could imitate him. He, as I know, would have studied these things, not by deduction, as from a law exactly formulated and from which he could trace the course of every change, but by a most careful collection of facts, facts to be seen in specimens and read in full records, and stored in museums, and by a study as complete for every case as if no law of evolution had ever been discovered.

Let me add that the study of these variations of diseases is not one of mere pathological curiosities. It may be of great practical utility; let me show how, if only that I may provoke some to pursue it vigorously to whom mere pathology is not attractive. We hear much, and often, of the uncertainty of medicines; of disappointments in the use of this or that supposed remedy; and substances which have long been in good repute for the treatment of this or that disease are spoken of with disrespect. It need not be questioned that in many cases the belief in the utility of medicine has been maintained by completely erroneous observations. Such was the belief in the utility of infinitesimally small doses of anything ever yet swallowed. And other beliefs less evidently absurd may have been nearly as ill-founded. But there are many of which this is not to be said. It cannot be doubted that bromide of potassium is often very useful in epilepsy; yet sometimes, as we say, it fails; or that guaiacum is useful in some cases of chronic rheumatic arthritis, and is in others very disappointing; or that arsenic sometimes does not do good in cases of lymphadenoma. I suppose there is not a medicine in the Pharmacopœia which does not sometimes disappoint him who gives it hopefully, not one which is not, therefore, spoken of with contempt or blame, as if it were a responsible agent convicted of default. But here is an unfair imputation. It is not these medicines which are in fault, but ourselves. That which some call the fallacy of therapeutics is generally the fallacy of diagnosis. To state the facts roughly, we suppose cases to be alike which are really different; and, very naturally, the medicine that does good in some of them is useless in others. For example, in the group of cases which I chiefly have in view, we do not always discern when a disease has varied so far from its usual type that it is no longer amenable to its usual remedies. A better diagnosis must precede a better therapeusis. We need not only the diagnosis between diseases essentially different, but that between the different and varying forms of each of those which we call by a generic name; and beyond this, we need a more exact power of what may be called analytic diagnosis; for there are few simple cases, and in those which are not simple we need to be able to discern all the components, and the proportions in which they are mingled or combined. Better treatment will follow better diagnosis, and better diagnosis will certainly follow a more exact pathology.

Let me illustrate this with an instance which is besides of some interest in the study of the variations of transmissible diseases and of the utility of museums. Questions are often asked as to the changes which syphilis may, in course of time, have undergone; and, especially, whether internal organs were always, as they are now, liable to its attacks. It is hard to answer such questions on the evidence of any existing records; indeed, I might cite the whole history of syphilis as an instance of the insuffiency of records for the tracing of the natural history of diseases. But here is something suggesting what museums may do; a portion of muscle preserved by Hunter, and at least a century old, in which are morbid changes which may be safely referred to syphilitic gumma. Probably similar evidence may be found in other museums, and there are other facts significant of the existence long ago of these internal syphilitic diseases, as well as the improved treatment following better diagnosis. Fifty years ago, at the beginning of my professional studies, it was the custom, as it long had been, to give mercury not only in all recognized syphilitic cases and in most acute inflammations, but in a large number of cases of which one could scarely say more than that they were all chronic and all obscure. Especially there were many such cases of what we considered chronic inflammation of the eyes, and of the brain and spinal marrow, the liver, and the testicle. To all of these cases it was customary to give mercury, till, as one said, "the mouth was touched," and thus some were cured, and some uncured, and some harmed. The cures were enough to keep the mercury in such good repute that it was given more and more generally; and then the disappointments, as they were called, became too many, and the mercury was blamed, and was almost disused for chronic inflammations. But, meantime, a more exact pathology, a pathology more exact both in its morbid anatomy and in its clinical studies, was discovering the previously unsuspected syphilitic diseases of internal organs; and with this better diagnosis a more judicious use of mercury, and good reason to believe that the chronic and obscure cases which mercury used to cure were those of syphilis overlooked. The case is an exemplary one of the relations between the true pathology and the right treatment of diseases, exemplary not only for encouragement, but for method of study; for the study was both clinical and anatomical, in the living and in the dead, with records and with specimens. Such must be our study of all the cases which I have chosen to speak of—the cases in which diseases deviate from their usual type, or combine in various proportions, after the manner of hybrids and mongrels or new chemical compounds. But there are some rules in study which are especially applicable to these cases.

1. We should very carefully study all cases which are not according to an admitted type. We should study all exceptions to rules; never thinking of them as unmeaning or accidental. Especially, we should never use, in its popular but wrong translation, the expression "exceptio probat regulam;" as if an exception to a rule could be evidence that the rule is right. If we use it, let this be in its real meaning; translating it, as surgeons should, that an exception probes a rule, tests it, searches it—as the Bible says we should "prove all things"—to its very boundary. In this true meaning the words may be an excellent motto for the study of all diseases that deviate from types.

2. We should look out for indications of the existence in the same person of two or more morbid conditions or dispositions such as may be derived from both parents or from several ancestors. For, as in plants and animals there are hyrbids and mongrels, or, as in chemistry, many compounds and mixtures, so are there in diseases. We see them in the multiform varieties of what we have to call rheumatic gout; in gout crossed with scrofula, or syphilis crossed or mingled with scrofula or with gout. It is often not difficult to discern some of these combinations among our cases, and I know few things in practice more useful than to be able, even in some intances, to adjust our treatment to the proportion of each disease in the compound. But we may be sure that there is much more to be learned in this direction; and it is best to believe that we rarely have to do with a simple and unmixed morbid constitution. There are few worse habits in practice than that of commonly saying of our case "It is all gout," and of another it is all scrofula, or all syphilis. We might as well say of any Englishman that he is all Norman, or all Anglo-Saxon, or all Celt. We may, indeed, sometimes see persons who appear to be as types of races unchanged in many centuries, but in practice we had better study every man as, for better for worse, a composite of many ancestors.

3. We should have for all these cases a much more complete and exact study of all the personal conditions of disease than is now usual. Of course, this should include all that can be learned of each patient's family history; though there are few parts of medical inquiry more fallacious than this often is; and at the best it will need, besides, the exactest study of the patient's self. Perhaps the brilliant success which has been achieved by the recent studies of disease-producing organisms or other materials acting on us from without—a success not equalled in any other field of medical inquiry—has made some think too little of those changes within ourselves which occur in such ordinary conditions of life that they may be called spontaneous. Yet these are not less important in the production of diseases, and these must be studied; just as in agriculture soils must be studied as well as seeds. This is true even in respect of those diseases whose essential causes are most evidently external, even of those which are due to specific contagia; their germs of seeds, if I may so speak, will not germinate in an unfit soil. I suppose there is not a day in which most of us do not inhale or come in contact with the germs of some frequent or contagious disease; but they do not germinate in us any more than do the seeds of tropical flowers in our streets or in the fields to which the wind scatters them; we do not offer the fitting soil. And even among those in whom they do germinate, the product varies according to the soil. And the study of this soil, this living soil, is yet more necessary in respect of diseases which come, in part or wholly by inheritance; for it is in each as personal and distinct as any other constituent of personal character, and the study of it must be intimately personal, with an exact analysis of every disposition to disease. The aim of pathologists in this direction should be for knowledge like that of the keen family practitioner, who, as he says, knows the constitution of every member of a family.

All this is equivalent to saying these variations in diseases mustbe studied both in practice and in scientific pathology. It is hopeless that either a practitioner who thinks lightly of pathology, or a pathologist who thinks lightly of observant practice, should do more in the study of these questions than attain to that measure of partial truth which is often as deceptive as error. Each must be tested by the other. The living and the dead must be alike and equally studied; and the dead must be studied in exact observations, with accurate records, and especially with museums.

I need not dwell on the vulue of good records, good descriptions, and good photographs, or other representations of diseases; but they never have been and, probably, never will be, enough. We need with them, museums, in which changes of structure may be preserved for repeated and revising study and comparison. For instance, in regard to the group of diseases of which I have been speaking, we ought to have in our museums specimens in which we might study all the gradations of change of structure from type to type, all the changes due to mingling of forms, all varieties of diseases, all hybrid forms. We need to be able to study all these things, and the naturalist or the comparative anatomist needs his specimens; not only for teaching what is known, but for continued re-examination and continued additions to his own knowledge.

And for complete study we must have large museums showing the coarse naked-eye characters of diseased structures. I am sure no one will think me likely to depreciate the microscope ; it has added, and will continue to add, more than can be told to our knowledge ; but it has not diminished the value of other evidence ; and in pathological anatomy, as in all our sciences, there are many instances in which the naked eye sees facts with more meaning than the microscopic one can.

This is especially true in the case of morbid structures resulting from nearly allied diseases, and therefore especially true for those of which I wish to urge the study. In morbid structures as in species, the nearer the alliance the less are the differences to be found in minute structures, and the more must we depend for distinctions on the study of visible shapes, and sizes, and constructions. I suppose that we could not with the microscope distinguish the human skeleton from that of the monkey ; certainly, we could not distinguish one skull from another in all those varieties of national form which are collected in our museum. And so it is in many instances of morbid bone formation. I doubt whether microscopic examination could detect characteristic differences in each of this group of specimens. With the naked eye it is sure that this is a syphilitic node on a tibia, and this a growth beneath a chronic ulcer over the shin, and this a pedicled exostosis, or ossified cartilaginous outgrowth from the shaft of a long bone, and this an instance of osteo-arthitis, and this a portion of the skeleton of an osteo-sarcoma or osteoid cancer.

Moreover, it is to be observed that in morbid structures, as in those that are natural, in the same proportion as the aggregated elements of embryonic structures acquire their complete and final form, so do the bodies composed of them acquire distinctive shapes and methods of construction plain to the unaided senses. The ova of many species may seem alike both in outer shape and in their component elemental structures. But im proportion as these structures are differentiated, and developed into their higher and abiding forms, as into nerve fiber, and muscular fiber, and the rest, so the larger characters of even the nearly allied species —the characters of shape, and size, and appropriate construction of the whole body, and of each part of it—become more and more different; and these constitute the real distinctive characters of each species.

And so it is in morbid products. The acquirement of distinctive shapes and methods of construction coincides with the development of elemental forms. For example, in these sarcomata are only the lowest elemental structures, round cells, spindle cells, and shapeless plasma; and the masses thus combined are shapeless, featureless, decisive by negation. But in these fatty, and fibrous, and cartilaginous, and bony tumors, in which the elemental structures have advanced to higher forms, the masses which they severally compose are almost as characteristic and distinct in visible shape and construction as are the several normal organs of the body.

In every case, then, both the largest and the smallest characters should be studied. The naked eye can discern one set of facts, the aided eye another; both are essential to complete knowledge; no one should be content with either, for neither is alone sufficient. So we must have large specimens as well as small ones, and certainly large ones for the study of the gradual variations of diseases as they deviate from typical forms and become variously mingled.

And now, as I come nearer to my term of time, let me, as is customary in certain other places, conclude with an earnest appeal to your liberality. We want liberal contributions, not of money, but of specimens to our museum. We want specimens of many kinds; of course we want whatever is rare, but not these alone; we want some to complete our series of typical specimens; and to keep to the chief subject of my lecture, we want the opportunity of choosing among many of what are called bad "specimens." We are all too ready to collect what are called good specimens as being well-marked instances of the standard characters of diseases, and to put aside as "bad" those which deviate from those characters, just as, clinically, we speak of good and bad cases of a disease. Of course, good specimens, typical specimens must be at hand for the teaching of pupils who have to study illustrations of the accepted descriptions of diseases; but it is among bad specimens, even as it may be among exceptional cases, that those who are past pupilage, though they have not ceased to be students, may study the variations of disease. I ask the more boldly for contributions to the pathological collection, because of its present satisfactory condition and the activity of work in it. You will soon see it in the repaired and renovated building. Looking at the number and value of the specimens and the wide range of pathology which they illustrate; looking at the interest of the history of our science which is told in many of them; at the memorials of Hunter and Matthew Baillie, of Astley Cooper, Liston, Howship, Lawrence, Hammick, Ferguson, Hilton and many more; looking forward to what the museum will tell of the researches and skill of those who are still with us, and among whose names I venture to feel sure, Mr. President, that none will take precedence of your own, while men study the specimens with which your skill and just audacity in operating have enriched the series of diseases of the ovaries and uterus: looking at all these things, and then at the perfect order and condition in which the specimens are preserved, I feel that the collection is one which all we members of the College may feel personal pride in calling our own, and should feel a personal duty to enrich. And its utility is being constantly more appreciated. I have been often made happy by the contrast which I have seen while working at the new edition of the catalogue. While I was writing the last edition between thirty and forty years ago, scarcely a student ever entered the museum. Hour after hour I sat alone; I seemed to be working for no one but myself, or for nothing but the general propriety that a museum ought to have a catalogue, though no one might ever care to study with it. Now, and for some time past, a day rarely passes without many pupils and others being at work in every part of the museum.

All this is good; but much more is to be done. Our museum should be, even more than it is, the center in which all pathologists may find help in searches after that which is not yet known; in such searches, for example as may lead to a complete knowledge of the variations of diseases. For many years, even from the beginning, the anatomical and physiological departments of our museum have been not only a noble collection of specimens, but, through the renown and learning of its conservators, a great center of teaching. Scientific men, especially comparative anatomists and anthropologists, have known that here, if anywhere, they could find whatever help a museum and a master in those sciences could give. A fortnight ago the president of the Royal Society, presenting one of the royal medals to Professor Flower, said: "Professor Flower has been for more than twenty years conservator of the museum of the Royal College of Surgeons; and it is very largely due to his incessant and well-directed labors that the museum at present contains the most complete, the best ordered, and the most accessible collection of vertebrate structures extant."

It is not for me to praise the pathological collection with similar words. But great as may have been its utility hitherto, we may be confident that it will henceforth be more useful than ever. In the vast increase of biological science it became impossible that one man should be nearly complete in the knowledge of both natural and pathological anatomy. I say impossible. I believe there is not such a one living; if there could have been one it might have been Mr. Flower. Now, we may hope as labors as "incessant and well directed" as his will be devoted specially to the pathological collection.

It is known to many of you that Sir Erasmus Wilson, in his usual liberality, gave the College £5,000, of which the interest should be spent in the promotion of pathology; and he agreed that this would best be done by helping to the appointment of a curator of the pathological department of the museum; and we have an admirable one. Mr. Eve is a worthy colleague and helper of Mr. Flower, excellent like him not only in knowledge, but in that which is even more rare, the love of museums, and of all that belongs to their maintenance and illustration, even to the making of catologues. In all these good qualities he has distinguished himself at St. Bartholomew's. I believe that we may rely on him for making as good use of the museum, and of all that can be brought to it, that the College shall be the chief center of pathology, even to the furthest point at which it can be studied in specimens of diseased structures. I beg your help that he may be so; and if I shall have helped to-day to this good result, the first Bradshawe lecture in our College will have fulfilled the purpose of its founder.

